# COUPLES’ VIEWS OF THEIR JOINT AGING: DEVELOPMENT OF A NEW SCALE

**DOI:** 10.1093/geroni/igae098.0551

**Published:** 2024-12-31

**Authors:** Ehud Bodner, Dikla Segel-Karpas, Roi Estlein

**Affiliations:** Bar-Ilan University, Ramat Gan, HaMerkaz, Israel; University of Haifa, Haifa, Hefa, Israel; University of Haifia, Haifa, Hefa, Israel

## Abstract

The construct of subjective views of aging (VoA) constitutes a growing field of research, relating to individuals’ general cognitions about the aging process, and their individual experience of aging. VoA develop throughout the life-course and have significant implications for individuals’ health and well-being in later age. Although VoA often develop within a relational context of the married couple, suggesting the construct has an inherent interpersonal component, no theoretical framework or empirical studies have explored VoA from a dyadic perspective to consider the couple as an aging unit. We developed and tested a new scale to assess the way individuals expect to age within their current intimate relationship. Based on experts’ evaluations, we identified five domains that are meaningful to couples’ joint aging: (1) changes in health, (2) changes in family roles, (3) the transition to retirement, (4) changes in appearance and sexuality, and (5) coping with death. We then developed an initial pool of 39 items and tested these on a sample of over 300 midlife adults. Using factor analysis, we extracted a scale of 9 items relating to the 5 domains. We tested for validity against constructs of VoA and couple’s relationships. The scale showed good internal reliability and validity.

